# Developing sustainable workplace through leadership: Perspectives of transformational leadership and of organizational citizenship behavior

**DOI:** 10.3389/fpsyg.2022.924091

**Published:** 2022-07-26

**Authors:** Cheng-Chung Cho, Rui-Hsin Kao

**Affiliations:** Department of Ocean and Border Management, National Quemoy University, Jincheng, Taiwan

**Keywords:** sustainable workplace, extra-role behavior of employees, human resource management, transformational leadership, organizational citizenship behavior

## Abstract

The objective of this study was to investigate the leadership style of the supervisor to develop the organization's sustainable workplace of and the extra-role behavior of employees (i.e., OCB). An organizational context of the immigration officer is explored by using the data collected from a survey of 453 immigration officers from 26 immigration officer teams in Taiwan. This study has verified the transformational leadership and organizational commitment that they have positive effect on organizational citizenship behavior (OCB) of the immigration officers. Moreover, it showed that the organizational climate has a context effect on organizational commitment and OCB. Furthermore, the results of this study have shown that an aggregated transformational leadership has cross-level interactions on OCB. This study also found that the transformational leadership has strong effect on organizational commitment and OCB. This study adopts a cross-level study taking organizational environmental factors and cross-level interactions as research considerations. Because of the focus on group-level impact, the research methodology can apply the supervisor's leadership style and the organizational climate to measure whether the immigration officers have a high degree of organizational commitment and influence their OCB performance across levels. The organizational commitment of the immigration officers and their individual OCB performance could be improved by converging the organizational context effect of aggregated transformational leadership and organizational climate. This study found that the application of the transformational leadership is extremely helpful for an organization to develop sustainable workplace and extra-role behavior of employees.

## Introduction

Kramar (2014) has pointed that the sustainable human resource management (SHRM) was a new way for the managers to identify the complexity of a dynamic workplace and determine the broader purpose of HRM. National Immigration Administration (NIA) of Republic of China is mainly responsible to manage the entry and exit of Chinese and foreigners, and the related foreigners' activities in Taiwan. Therefore, it is important to improve the quality of service for the immigration officers by developing sustainable work, workplace, and the extra*-*role behavior of employees through the use of appropriate leadership styles. Because the subjects of the immigration officer service are the general public, the quality of immigration officer service is evaluated by the people who receive the service. Grönroos (1984) attempted to illustrate from the point of view of interaction theory that the quality service is determined by the shared experience and care of customers and service personnel involved in the service. Therefore, it is very important for the immigration officers who directly contact with Chinese and foreign tourists to provide the high-quality services. In addition, leadership is a process that determines whether an organization can remain successful in the face of difficulties and in competitions. Good leaders not only unleash the potential of their employees to be more productive (Mach et al., 2022) but also meet their needs and achieve the organization's goals. In fact, the leadership plays a key role in shaping the behavior of the immigration officers (Chang et al., 2021; Shofiyuddin et al., 2021). Therefore, the leadership of immigration officers has always been at the heart of the search for the best management of immigration officer (Silvestri, 2007). The leadership style and characteristics of leaders, and the essence of what leaders should behave and cultivate, have long been widely discussed in many studies (Men, 2014). For example, Anwar et al. (2020) pointed out that the leadership style of the principal has affected on the discipline of teachers. The principal should be more informed in deciding on leadership style and making it easier for teacher to perform the teaching task to achieve the school's goals. Ma et al. (2020) also showed that the leadership characteristics of leaders play an important role in improving the effectiveness of the employees. Furthermore, in any successful organization, the leadership plays a key role (Dube et al., 2022). Therefore, the argument for a transformational leadership has been a topic of interest (Kao, 2017).

Research has shown that the climate of workplace can significantly affect the individuals and their organizations (Jia et al., 2008). Some examples are work attitudes and satisfaction (e.g., Judge et al., 2017), service quality (e.g., Weller et al., 2020), and customer satisfaction (e.g., Kloutsiniotis and Mihail, 2018). As a result, in an early empirical study of Likert (1967), organizational climate had been seen as an important basis for interpreting and analyzing organizational phenomena (Gajić et al., 2021).

Furthermore, there are many concepts to describe the relationship between organizations and their employees. Two major employee attitudes are organizational commitment and organizational citizenship behavior (OCB). For example, organizational commitment is associated with several variables of organizational outcomes, including absenteeism (e.g., Jacobsen and Fjeldbraaten, 2020), turnover intention (e.g., de la Torre-Ruiz et al., 2019), work satisfaction (e.g., Massoudi et al., 2020), person–organization value fit (Zhao et al., 2021), and innovation performance (e.g., Iqbal et al., 2021). For non-profit service providers such as immigration agencies, fire agencies, and public libraries, they essentially provide free service to their clients (Talaga, 2008) due to their altruistic nature. Relevant studies had shown that the organizational commitment and OCB could improve the organizational performance, especially the reveal of service quality (Heydari and Lai, 2019). Therefore, there is a tendency to study the organizational commitment, OCB, and other relevant variables (Op den Buijs et al., 2019). Yet, compared to the private agencies, there is little or no research on the organizational commitment of immigration officer (Hidayati and Sunaryo, 2019) or OCB (Kao, 2017).

This study has not only applied transformational leadership as a variable at individual level but also at group level (i.e., aggregated transformational leadership, ATL). Therefore, in addition to exploring the personal perspective on the type of leadership, this study has also focused on how the leaders could approach their groups (Arendt et al., 2019). Moreover, the study also viewed organizational climate as a variable at organization level. The different organizations have the different cultural characteristics and work conditions. Therefore, the organizational climate is a social cognitive response of an individual toward his/her organizational environment and can vary significantly among organizations. As a result, how organizational climate is perceived by employees is pluralistic and can lead to different types of employee behavior (Wangombe et al., 2014). Last but not the least, organizational climate has a strong contextual effect (Kao, 2017).

Taken together, the purpose of this study was to investigate transformational leadership to develop organizational sustainability in the workplace and employees' extra-role behaviors. This study has applied cross-level study to incorporate the organizational contextual factors and cross-level interactions into a theoretical model for discussion. The research method takes the group-level effects into account and is applicable to cross-level immigration agencies (such as corps, brigade, and squad). It could measure how a leader's leadership style and the organizational climate of the team determine whether an immigration officer has a high level of OCB. At the same time, through the contextual effects of aggregated transformational leadership and sharing of organizational climate, the member's common perception of organizational leadership style and organizational climate can improve the individual organizational commitment and affect the reveal of individual OCB.

## Literature review and hypotheses development

### Literature review

#### Transformational leadership

Any organization needs a management, and the management, in turn, needs an unquestionable level of leadership capacity (Wu, 2009). Brazier (2005) pointed out that a leadership is the process by which an individual influences a group of people to achieve a common goal. A transformational leadership values the motivation of the members, and gives subordinates a long-term perspective (Ronald and Marc, 2021). The issue of leadership has been widely valued and studied by the researchers in the past. For example, Schuckert et al. (2018) believed that the transformational leaders can use intellectual simulation to encourage subordinates to view things from different perspectives, and proposed individualized consideration to motivate and support subordinates, and drive inspiring motivation to express the vision of the organization. At the same time, it can highlight the charm of the leader and stimulate the subordinates' feelings and recognition of the organization and leadership. The transformational leaders could demonstrate a leader's consideration for the individual employees by listening attentively, observing the performance of subordinates (such as coaches or supervisors), and meeting the developmental needs of the subordinates (Kim and Lee, 2021). The transformational leadership would motivate the employees to reveal their greatest potential by encouraging them to take more responsibility (Bose et al., 2021), and point out that the employees should value the vision of the group interests (Li et al., 2016). These would be the unique characteristics of the transformational leadership.

In this study, the investigators adopted transformational leadership as a type of leadership of immigration officer because of the following reasons. First, Bass and Avolio (1993) clearly stated that the concepts of the transformational leadership (including treating charisma as the main factor) are appropriate for the leadership at all levels as specified as follows: From the individual-level leadership (or leadership of small groups) to the leadership for large organizations and to meta-leadership (leadership of movements and societies). Second, over the past two decades, the transformational leadership theory has been popular in leadership research because from the perspective of subordinate monitoring (Mansoor et al., 2021a). To compare with other leadership styles, the transformational leadership employs a different approach to motivating the subordinates, such as providing them with a long-term organization vision. Through the communication of the aforementioned organizational vision, the subordinates can feel that the organization value themselves, which, in turn, makes them work harder, thereby improving their participation and job satisfaction with the organization (Moreira Mero et al., 2020; Nemteanu and Dabija, 2021). Moreover, by focusing on the employees and making them feel supported, the leaders can strengthen the competitiveness of their organizations (Nemteanu et al., 2021). Because serving the public is one of the core values of an immigration officer in Taiwan, he/she has to display altruistic behavior and possess a high moral value and standard for serving the public. Research (e.g., Hoch et al., 2018) has shown that a great proportion of emphasis of transformational leadership has been placed on employees' moral behavior, and transformational leadership style is featured by moral righteousness. Last, there were many studies of the immigration organizations. For example, Adebayo (2005) and Rowe (2006) had found that the transformational leadership displays knowledge and skills suitable for the organization, elevated organizational commitment of immigration officer, and promoted the expression of citizenship behavior of immigration officer. The result is that the leadership is beneficial for elevating the quality of immigration officer service. We can say that the harbor immigration organizations require a leadership that not only provides employees a vision but also stimulates their altruistic behavior for the general public.

#### Organizational climate

Organizational “climate” is an approach for people to learn about their work environment (Verbeke et al., 1998). It is also an organizational feature that is relatively stable because it cannot be altered rapidly or easily (Denison, 1996). The climate represents a perceptual pattern or thematic experience of an employee, which is the conceptualization of an organization member's experience of their workplace (Yang and Caughlin, 2017). Because climate is subjective, short-term and hidden, it can be easily manipulated by the management (Denison, 1996). Organizational climate is a group of individuals who have a direct perception of their work and life in their workplace, and which can evaluate the look of the work environment (Litwin and Stringer, 1974). Based on employees' common perception on the workplace climate, an organizational climate is believed to have an impact on the employees' motivation and behavior.

To clearly differentiate between the different conceptual levels of organizational climate, James and Jones (1974) indicated that the organizational climate employees' individual environmental perception is a type of psychological climate, whereas organizational climate itself is a combination of those perceived by individuals on the group level or on the organizational level. Once all employees of a unique work unit perceive their workplace environment similarly, this shared perception would aggregate and become an organizational climate shared by the members (Moslehpour et al., 2019; Martinolli et al., 2021). Therefore, the climate structures share common content, meaning, and structural validity across different levels of information aggregation (Elgesem et al., 2015). This feature makes the organizational climate theory important when the cross levels of an organization is studied (Schneider et al., 2013).

Taken together, the environmental perception is involved in organizational climate, and organizational climate is related to how the daily business handled by the employees of an organization is perceived by the employees consciously (Kao, 2017).

#### Organizational commitment

Organizational commitment is a type of psychological association between an organization and its employees (Sudiro et al., 2021). It is also a type of employee spiritual force that makes the organization work (Asutay et al., 2021). Some studies have discussed the organizational behavior variables related to organizational commitment. For example, in the study on “influence of internal marketing dimensions on organizational commitment,” Moreira Mero et al. (2020) found that the leaders and managers can strengthen their subordinates' organizational commitment through policies that encourage employee motivation and satisfaction, and contribute to achieve organizational goals. Moreover, in the study on “how to use human resource approaches to promote the motivation of public employees,” Ciobanu et al. (2019) found that through high-commitment HR practices, the employee commitment to the organization and work can be promoted, and the job performance can be improved. Relevant studies found that the employees with high commitment are more capable to make positive contributions to the organization than those with low commitment (Zhu and Song, 2022). Therefore, a high degree of organizational commitment could be regarded as a kind of positive factor in shaping the advantages of organization and work environment (Rujit and Liemsuwan, 2021).

For the organizational commitment aspect, Mowday et al. (1982) formulated an organization commitment questionnaire for evaluating the employee organizational commitment. They classified organizational commitment into the following three variables: (1) *Value commitment:* It is a type of psychological state in which the employees go from believing in the organization to accepting the goals and values set by the organization. (2) *Effort commitment:* It is a psychological intention of employees to achieve organizational goals. (3) *Retaining commitment:* It is about the willingness of employees to look forward to staying in the organization. Organizational commitment represents the identification of the employees with the organization and their willingness to give loyalty to the organization. It is a continuous, normative, and emotional commitment of employees to the organization (Khan et al., 2021). It goes beyond the differences between the existing attitude and behavioral commitment (Meyer and Allen, 1991). Meyer and Allen (1991) pointed out that the commitment would reflect at least three different factors, including the employee's (a) desire (affective commitment), (b) need (continuance commitment), and (c) obligation (normative commitment) as a state of mind to continue working in the same organization. Moreover, Metcalfe and Dick (2002) developed a commitment measure for the immigration organizations that included pride, goal, and involvement factors. In addition, there are three following dimensions of organizational commitment: Affective commitment, normative commitment, and continuance commitment (Khan et al., 2021). As mentioned above, the organizational commitment is the mental state of members who accept the values and goals of the organization, work hard, and retain the job position in the organization. There were few studies that investigated the organizational commitment of immigration officer.

Van Maanen (1975) was the first scholar to study the development trend of organizational commitment. The results have shown that the organizational commitment of immigration officers would decrease as experience and seniority increase. It may be due to the strong characteristics of the socialization process of immigration officials. Some Australian empirical studies, including the immigration officer Service in New South Wales, indicated that the organizational commitment of immigration officers was low compared to the international standards (Ma et al., 2020). Therefore, the importance of management factors in the development of organizational commitment levels could be recognized from the study of immigration officers (Veličković et al., 2014). Currie and Dollery (2006) examined 351 officers in New Zealand and revealed a low organizational commitment, independent of gender, external capabilities or types of responsibilities. Nonetheless, they found that the organizational commitment drops with increased seniority (while age increases and job position elevates). Therefore, a critical factor of the performance of the immigration officer lies in the level of commitment of individual officer to their responsibilities (Currie and Dollery, 2006).

#### Organizational citizenship behavior

In the study about organizational behavior, OCB was first proposed in the 1980's (Ocampo et al., 2018). It has been generally regarded as an individual altruistic behavior or an unconditional working behavior of employees (Thöni et al., 2021). Furthermore, OCB has been defined as a type of behavior that could help clients, colleagues, or their leaders (Deery et al., 2017). Since it was not an expected behavior of employees, it would not be included in the employment contract (Kao, 2017). In the relevant study of OCB, it has been considered that it could be a type of unconditional behavior of individuals. It could not be specifically or directly recognized through the reward system of the organization, and it could not improve the effectiveness of organizational operation (Nadeem et al., 2019). Furthermore, the focus of OCB is on confirming employee behavior. Even though there is no definite definition of employee behavior in most work manuals, OCB can still elevate the organization efficacy (He and Kim, 2021).

In short, OCB is an act that can be initiated voluntarily by an individual without the request of others or organizations and may affect customer satisfaction by acting in a manner that maximizes the interests of the organization. Many OCB studies have found that it could make a special contribution to the organization and has a significant contribution to the improvement of the quality of the organization's service (Yuan et al., 2020). Through the OCB presentation, employees could provide higher quality service that exceed the formal expectation of their organization (Hongbo et al., 2021). The main task of NIA is to manage the activities of foreigners in Taiwan, and the management of the entry and exit of Taiwanese and foreign passengers (Elche et al., 2020). In summary, how to improve the quality of service of the immigration officers is important to ensure that they are thoroughly on duty to maintain NIA's border management responsibilities. Furthermore, the incorrect work attitude would expose the organization to a negative perception (Brown et al., 2020).

The study of the immigration officers should focus on the OCB of immigration officers. On the other hand, because of the cultural differences among countries, OCB of Western world may differ from that of Chinese world. As a result, many researchers have examined OCB implications in Chinese organizations and proposed OCB dimensions that are different from Western researchers' findings. For example, Farh et al. studied Chinese in Taiwan and Mainland China in 1997, 2000, and 2004 by observing Chinese employees OCB and collecting the data for developing an OCB inventory specifically for employees in a Chinese society (Farh et al., 1997). It can be found from the OCB dimensions of the three studies that some of them are quite similar to what were proposed by study of Organ (1988), including civic virtue, altruism, and conscientiousness. As for protecting and saving company resources proposed by the three studies on Chinese, they were not part of the OCB dimensions of the Western society. As a result, cultural differences could affect the content of OCB dimensions. Lin and Peng (2010) adopted a social exchange theory perspective for examining Chinese organizations and found that Chinese OCB content should include in-role behavior, organizational behavior of public interest, and interpersonal altruism in a concurrent way. In other words, employees displaying OCB should exhibit work behavior expected for the role they play, take care of organizational benefits, be willing to assist the colleagues actively, and care about the behavior of the colleagues.

## Hypotheses development

### Relationship between the variables in this study

#### Association between group-level and individual-level variables

The associations between climate and different organizational outcomes have been well-documented in the literatures (Kao, 2017; Caniëls and Baaten, 2019). Existing studies revealed that at individual and group levels, transformational leadership has highly positive correlation on work attitudes and behaviors (Ge et al., 2022). Some studies also explored the role of leaders in management, such as organizational outcomes related to the organizational innovation (Alblooshi et al., 2020), organizational climate (Bartsch et al., 2020), organizational commitment (Ma et al., 2020; Kawiana et al., 2021), and OCB (Moon, 2016). Besides, the study has also tested the relationship between the management style of transformational leadership and the atmosphere of innovation support. They are found to be significant positive correlation (Kohan et al., 2018; Mansoor et al., 2021b). The aforementioned results have shown that the leadership style should be combined with the organizational climate so that the leadership style and the organizational climate could complement each other and make the organization work smoothly. In addition, the study also revealed that the supervisor's support can have a positive impact on the official's organizational commitment (Ge et al., 2022). In conclusion, if the leaders and their employees can build a good working relationship, this relationship can lead to a strong organizational climate while increasing the organizational commitment (Luthans et al., 2021). In addition, the previous studies have found (e.g., Alkahtani, 2015; Von Treuer et al., 2018) the organizational climate variables, such as autonomy and cohesiveness are positively related to organizational commitment. On the other hand, transformational leaders have incentivized subordinates to implement OCB that exceed the expectation (Novianti, 2021). Ardi et al. (2020) claimed that when officials recognize that their leaders are highly change-oriented, they are encouraged more than expected than they would be to those who are less change-oriented.

At the individual level, employee satisfaction, organizational commitment, and recognition on just and support from leaders have been statistically identified as LEADING variables of OCB (Eliyana and Ma'arif, 2019; Djaelani et al., 2021). In addition, organizational climate and organizational commitment studies also found that OCB has a significantly positive effect (Zhao et al., 2022). Therefore, at the group level, this study believed that an aggregated transformational leadership may positively affect the organizational climate. At the individual level, the transformational leadership may affect the organizational commitment and OCB positively. In addition, the organizational commitment may play a mediating role between the transformational leadership and the OCB relationship, and the organizational commitment may have positive effect on immigration officers' OCB representation. Therefore, the investigators formulated Hypotheses H1, H2, H3a, and H3b as follows.

*H1*. Aggregated transformational leadership has positive effect on organizational climate.
*H2*. Transformational leadership has positive effect on organizational commitment.
*H3a*. Organizational commitment has positive effect on organizational citizenship behavior (OCB).
*H3b*. Organizational commitment has a mediating role played for transformational leadership and organizational citizenship behavior (OCB).

#### Cross-level effects of aggregated transformational leadership and organizational climate on individual-level variables

In the multi-level organizational theory model, the variables of group level would affect the dependent variables of individual level across levels (Li et al., 2013). That is, an aggregated transformational leadership not only affect the organizational climate but also affect the organizational commitment of an individual and reveal of the OCB across levels (Khaola and Rambe, 2020). Thus, the study on leadership must consider the correlations between variables at different levels. The existing studies have shown that the transformational leadership could affect the dependent variables such as intention of resignation and OCB (Virgiawan et al., 2021), and could affect the organizational contextual variables (Saira et al., 2021). Therefore, through multi-level research, employees' perceptions of leaders can be aggregated to the group level and their relationship with organizational commitment can be tested (Tremblay et al., 2019). This study has defined transformational leadership as individual-level and group-level variables, rather than just individual-level variables. Therefore, the attributes of a transformational leadership make it a contextual variable, by which through individual level of explanatory variables, it can be achieved to aggregate high-level of explanatory variables (Portoghese et al., 2015).

According to statistics, the effects of contextual variables are the aggregation of the mean of individual-level variables to high level. After the contextual variables are formed, their effect of the intercept or slope could be detected (Heisig and Schaeffer, 2019). The study by Chen and Kao (2011) has shown that by using a multi-level model, the variables of group level could be applicable to test the dependent variables of individual level. Thus, this study has proposed two cross-level direct effect hypotheses in sequence as follows:

*H4a*. Aggregated transformational leadership has positive effect on individual-level organizational commitment.
*H4b*. Aggregated transformational leadership has positive effect on individual-level OCB.

Similarly, an organizational climate also influences an individual-level organizational commitment and OCB. For example, Hung et al. (2018) found that the organizational climate has a positive effect on employees' organizational commitment, and influences the employee's willingness to leave through the mediating role of the organizational commitment. In addition, Shbail and Shbail (2020) pointed out that organizational climate is an important antecedent for employees to be able to demonstrate OCB. When employees of a same work unit reach consensus on their work condition influence, a common perception is aggregated into the organizational climate. In other words, the organizational climate is assessing how influences an individual perceive from the work condition (Kao, 2017). This study has defined organizational climate as a group-level variable. Since this study would apply cross-level analysis, hypotheses on the effect of group-level on individual-level dependent variables could be tested.

In summary, the organizational climate could affect various organizational outcomes such as organizational commitment (Berberoglu, 2018) and OCB (Shbail and Shbail, 2020). Therefore, two hypotheses about cross-level direct effects have been proposed as follows:

*H5a:* Organizational climate has positive effect on individual-level organizational commitment.
*H5b:* Organizational climate has positive effect on individual-level OCB.

#### Cross-level moderating effects of transformational leadership and organizational climate on individual level of dependent variable

Since the objective of this study has been primarily examining the effects of transformational leadership and organizational climate on organizational commitment and OCB using a multi-level model, the individual-level organizational commitment will be treated as a mediating variable, while the group-level contextual variable can generate a cross-level moderating effect on the individual outcome variables. In other words, the combination of explanatory variables at group level and at individual level may have cross-level effects on dependent variable. For example, Hsu and Chen (2017) found that there was a direct impact of organizational innovation climate on employee innovative behavior through cross-level research. The investigators explained the principles and assumptions of a multi-level model. Relevant studies have shown that in multi-level model, in addition to affecting the individual level of variables, the group-level variables may also affect how the individual-level variables explain the dependent variables. This situation could also call interaction (Kao, 2017). From the point of view of statistics, the group-level variable would act as a moderator, and would affect the explanatory power of the individual level of explanatory variable to the dependent variable. This is called slope effect (Li et al., 2013). Martinez et al. (2020) applied a cross-level research method and found that the transformational leadership can directly affect the organizational behavior of employees, such as work engagement. In other words, ATL and organizational climate (denoted as *Z*_1_ and *Z*_2_) not only directly affect the OCB (denoted as Y) but also interact with the organizational commitment (denoted as X), and therefore indirectly affect the OCB of the immigration officer. This effect can be presented by the following mixed equation:


(1) $$\begin{array}{r} Y_{ij}=\gamma_{00}+\gamma_{10}X_{ij}+\gamma_{01}Z_{j}+\gamma_{11}Z_{j}X_{ij}+u_{0j}+u_{1j}X_{ij}+\varepsilon_{ij} \end{array}$$

In the equation, γ_01_ is the effect (slope) of aggregated transformational leadership or organizational climate, and organizational commitment. γ_10_ means the effect (slope) of organizational commitment, and γ_11_ is effect (slope) of Z^*^X. This reflects the intensity of the impact on the OCB of immigration officers when combined with an integrated transformative leadership or organizational climate combined with organizational commitment. That is, it indicates the slope of the second level variable that explains the slope of first level. It could be called cross-level moderating effect and could be interaction effect. For example, Song et al. (2020) used the HLM statistical method in their cross-level study, taking the transformational leadership as the slope of the second level, the transformational leadership can mitigate the effects of prohibitive voice on self-efficacy across levels. Methodologically, the cross-level interaction reveals the multiple functions of group-level variables. In addition to directly influencing the dependent variables, it also strengthens (or weakens) the explanatory power of individual-level variables. For example, Martinez et al. (2020) found in the study on the cross-level moderating effect of transformational leadership, the team's shared perception of leadership can interact with the employee's emotional needs for the employee engagement.

According to the research design of this study, the impacts from two group-level explanatory variables (transformational leadership *Z*_2_ and organizational climate *Z*_2_) can act on the individual-level OCB *via* two cross-level interactions ($Z_{1}^{*}$X and $Z_{2}^{*}$X). Therefore, the investigators proposed two hypotheses on the cross-level moderating effect:

*H6*. There is an interactive effect from aggregated transformational leadership and individual-level organizational commitment on the OCB.
*H7*. There is an interactive effect from the organizational climate and the individual-level organizational commitment on the OCB.

## Methodology

### Framework of this research

**Figure 1:**
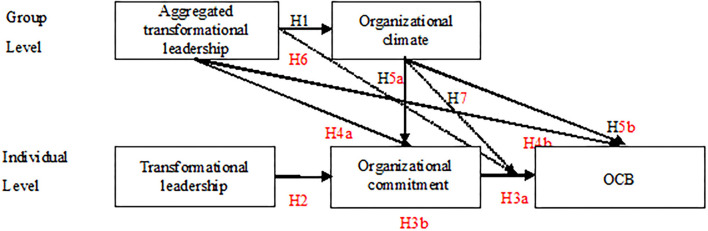
Research model.

The theoretical model of this study has been shown in Figure 1.

### Analysis strategies and the sample

#### Data test method

First, to test the hypotheses of this study, the confirmatory factor analysis (CFA) has been applied to test the construct validity of each research variable. Second, the structural equation modeling (SEM) has been applied to explore the relationship between the variables of individual level, and to explore whether there was mediating mechanism in organizational commitment. Furthermore, the hierarchical regression has been applied to analyze the relationship between the group-level variables to understand the effect of aggregated transformational leadership on organizational climate. Finally, the cross-level direct influence and interaction of group-level variables on individual-level outcome variables are analyzed by hierarchical linear regression mode (HLM).

#### Sample

The sample consists of a total of 453 immigration officers from 26 teams of Taiwan. The average of each immigration team is 17.32 immigration officers (range, 12–40). Each immigration officer is treated as a sample unit. The data collection is conducted in monthly immigration training congregation throughout the year. Among the 523 samples, 453 are valid samples. For the immigration teams, the average response rate to the questionnaire was 80–90%, and the overall average was 86.62%. The average age of the subjects was around 37.19 years and the seniority of the immigration officer was 11.21 years. Among all the subjects, 83.1% of them were male, and 82% of them came from the first-line policemen. This is due to the fact that most of the first-line immigration officers are transferred by the foreign affairs police in Taiwan. Other information includes that the profiles of the subjects are education, position, and service unit.

#### Measures of research variables

##### Transformational leadership

Transformational leadership was measured by using 19 items which was modified from multifactor leadership questionnaire (MLQ) developed by Bass and Avolio (1997), including idealized influence (six items), inspirational motivation (five items), intellectual stimulation (four items), and individualized consideration (four items). The MLQ has been extensively used and is considered a well-validated measure of transformational leadership (Kao, 2017). Its construct validity has been demonstrated using CFA (e.g., Boamah and Tremblay, 2019). All four theoretically distinctive behavioral components of transformational leadership were operationalized in the MLQ (Shofiyuddin et al., 2021).

##### Organizational climate

Organizational climate was measured with scales by modifying Kao (2017) which was based on Litwin and Stringer's (1974) Organizational Climate Questionnaire (LSOCQ). There were nine items contained in the scale. It included interpersonal relationship (four items), structure climate (three items), and responsibility climate (two items). Kao (2017) cited the above two scales in the study of Taiwan police organization and obtained good confidence values. For example, the value of transformational leadership was 0.902 and the value of organizational climate was 0.808, and they had good construct validity.

This study has measured the scores of the group. Taking organizational climate as an example, the scores have been calculated from the three scales, structural climate, interpersonal relationship, and responsibility climate. After adding up the scores of the three subscales, the organizational climate scores of the individual have been obtained. Then, the scores of each individual were put into the collective composition model to obtain an average value, and it was used as the organizational climate scores of that group. To estimate the overall variance of the variables, the proportions can be interpreted by the group differences. This study has applied ICC (1) to measure the differences between the groups (Biemann et al., 2012). The aggregated transformational leadership has also been calculated in the same way (Mehta and Mehta, 2018).

##### Organizational commitment

This study applied the questionnaire developed by Porter et al. (1979) and made relevant modification to measure the organizational commitment of the subjects. The scale consists of three factors, and a total of 14 questions; the values of alpha reliability of the questionnaire are 0.78 (value), 0.71 (effort), and 0.82 (retention). This study has also applied CFA to measure the construct validity of organizational commitment. The scores of subjects on the aforementioned three dimensions were averaged into a single index of organizational commitment.

##### Organizational citizenship behavior

This study has applied the research questionnaire of MacKenzie et al. (1993) to measure the organizational citizenship behavior of the subjects. The scale was modified from the OCB scale developed by Organ (1988). The OCB scale was divided into four dimensions and a total of 12 questions; the values of alpha reliability values of the questionnaire are 0.87 (sportsmanship), 0.76 (civic morality), 0.81 (conscientiousness), and 0.83 (altruism). This study has applied CFA to measure the construct validity of OCB. The scores of subjects on the aforementioned four dimensions were averaged into a single index of OCB.

##### Control variables

There may be many confounding variables that affect the relationship between variables in the group-level and individual-level variable analyses. In group level, the number of people would affect the group interaction and the operation way of the organization (Heisig and Schaeffer, 2019). Therefore, this may also affect how employees reveal OCB (Chen and Kao, 2011). Thus, the size of the subject group was used as the control variable. At the individual level, the study found that the amount of time the employees join a group affects how often they interact with each other, which, in turn, affects their behavior (Kao, 2017). Therefore, the working seniority of the subjects was also included as a control variable in this study. Besides, the education level and age of the members would also affect the information of the questionnaire (Ma et al., 2020). Therefore, they were also included as the control variables.

#### Data collection and informed consent

This article gave informed consent to let the subjects fully understand the purpose and procedures of this study. The subjects filled out the questionnaire at the sighted site and retracted it as soon as it was completed to increase the completion rate. They were answered anonymously so that the responses of the subjects were kept confidential. The information is obtained based on the answers to the questionnaire of the voluntary respondents.

## Result

### Basic analysis

After correlation analysis, each demographic variable has no correlation with other research variables; so, it is not introduced into the research model of statistical testing. The values of the correlation coefficient, α, SD, and mean between the variables of this study could be found in Table 1. This study performed CFA and compared the three individual-level potential constructs of the transformational leadership, organizational commitment, and OCB by maximum likelihood of LISREL to explore whether these three variables were different potential constructs. The CFA test results of the transformational leadership, the organizational commitment, and the OCB could be found from Table 2, which showed that they were of different potential constructs.

**Table 1:** Descriptive statistics and intercorrelation, and alpha reliabilities.

**Variable**	***M***	***SD***	α **coefficient**	**Research variables**								
				**1**	**2**	**3**	**4**	**5**	**6**	**7**	**8**	
(1) Transformational leadership	3.79	0.56	0.93	1								
(2) Organizational commitment	3.68	0.44	0.82	0.49^***^	1							
(3) OCB	3.44	0.48	0.80	0.57^***^	0.66^***^	1						
(4) ATL (group level)	3.94	0.36	0.95	−0.02	0.11	0.03	1					
(5) Organizational climate (group level)	3.51	0.29	0.88	−0.03	0.10	−0.07	0.44^**^	1				
(6) Age (years)	37.19	1.43		0.04	0.09	0.04	0.02	0.08	1			
(7) Education levels (years)	15.38	2.11		0.05	0.03	0.08	0.08	0.04	−0.21^*^	1		
(8) Years of service (years)	11.21	3.14		0.01	0.08	0.06	0.06	0.03	0.24^*^	0.04	1	
(9) Size of the group (person)	17.32	3.89		0.12	0.11	0.09	0.05	0.12	0.03	0.08	0.04	1

* *p < 0. 05;*

** *p < 0.01;*

*** *p < 0.001*.

**Table 2:** Goodness of fit statistics of the individual-level variables.

**Research variable**	χ^2^**/*****df***		**GFI**		**NNFI**		**PGFI**		**RMSEA**	
	**Observed value**	**Ideal value**	**Observed value**	**Ideal value**	**Observed value**	**Ideal value**	**Observed value**	**Ideal value**	**Observed value**	**Ideal value**
Transformational leadership	2.15	1.00~	0.96	>0.9	0.97	>0.9	0.63	≥0.5	0.066	* ≤* 0.08
Organizational commitment	2.71	5.00	0.94		0.93		0.59		0.071	
OCB	3.85		0.92		0.92		0.55		0.078	
References	Schumacker and Lomax (1996)		Bagozzi and Yi (1988)						Baumgartner and Homburg (1996)	

The main emphasis of the study was on HLM analysis, in which the investigators adopted the grand-mean centering approach for handling all the explanatory variables of the second level because this approach is good at reducing the covariance between the slope and intercept. By doing so, the potential multicollinearity problems can be minimized (Hofmann, 1997).

### Aggregated data testing

In this study, to test whether the transformational leadership and organizational climate could be regarded as the variables of group level, the correlation coefficients (ICC-1 and ICC-2) in the group has been applied to the test. This approach complies with the statistical principles (Biemann et al., 2012). For ICC-1, the score from each participant was used to compare the variance between analysis units (e.g., bank branches). The variance within each analysis unit, ICC-2, on the other hand, was about applying the average score of each participant of a same unit to evaluate the correlation status of the between-group as well as the within-group variabilities (Kao, 2017). The test found that the ICC-1 of the transformational leadership and the organizational climate were 0.21 and 0.26, while they were 0.91 and 0.72 on ICC-2. The ICC-1 coefficient between 0.05 and 0.30 would meet the criteria (Mehta and Mehta, 2018) while the critical value for ICC-2 coefficient should be above 0.60 (Martinez et al., 2020).

In summary, both ICC-1 and ICC-2 of the group variables met the criteria and were significant in this study. In addition, the test results showed that the *F*-value of the transformational leadership and the organizational climate reached significant. It indicated that that both variables had group effect. The detection values for the transformative leadership and the organizational climate were η^2^=0.301, *F* = 7.89, *p* < 0.001 and η^2^=0.201, *F* = 5.622, *p* < 0.001, respectively. This study has also referred to the practice of Chang et al. (2017) to count the *r*_wg_ values of the transformational leadership and the organizational climate to further test the appropriateness of their aggregation. The *r*_wg_ values of transformational leadership and organizational climate were 0.90 and 0.86, respectively, which met the criteria that the *r*_wg_ value should be higher than the critical value of 0.70 (Sheehan et al., 2020).

### Hypotheses testing

#### Hierarchical regression analyses

To test the effect of the control variables at the group level on the aggregated transformation leadership and organizational climate, this study has applied hierarchical regression analysis (HRA) to test the procedure. According to model 2, as listed in Table 3, it could be observed that the *F*-value of the aggregated transformation leadership has reached the significant level and β = 0.299, *p* < 0.001, and Δ*R*^2^ = 0.242. This statistical result has shown that the aggregated transformational leadership had strong explanatory effect on the organizational climate. Therefore, H1 was supported. In addition, according to Table 3, it could be observed that control variables of individual level, such as age, education background, and seniority have no significant effect on organizational commitment and OCB. Moreover, the number of group members had no significant relationship with organizational climate either.

**Table 3:** Hierarchical regression analysis.

**Model independent variables (group level)**	**Model number**			
	**1**	**2**	**3**	**4**
Group size (control variables)	0.019	0.007		
Transformational leadership (independent variable)		0.299***		
*F*	0.211	17.891***		
Adj. *R*^2^	0.010	0.242		
Model independent variables (individual-level control variables)				
Ag			0.038	0.078
Education level			0.025	0.049
Years of service			0.042	0.058
*F*			0.517	0.614
Adj. *R*^2^			−0.014	0.016

*Dependent variable: Model 1 and 2 are for organizational climate; model 3 for organizational commitment; model 4 for individual OCB*.

*The bold values indicate the test indicators for each hypothesis*.

The *** symbol indicates the value of p < 0.001.

#### The SEM test

The SEM was adopted to test the overall goodness-of-fit of the individual-level model. This study has applied LISREL 8.52 for the test. In the analysis, the error variances and factor loading values were calibrated by the measured variances and internal consistency reliability. The questionnaire scores were used as the single guideline for calibrating measurement errors and individual construct (Kao, 2017). As shown in Table 2, the individual-level variables of the overall hypothetical model were χ^2^/*df* = 3.27, GFI = 0.94, NNFI = 0.93, PGFI = 0.62, and RMSEA = 0.069. The results showed that the goodness-of-fit of this model was good. In addition, according to the statistical results, it could be obtained that there were significant relationships between the transformational leadership and organizational commitment; and transformational leadership and OCB. Their correlation coefficients were γ = 0.68, *t* = 5.07, *p* < 0.001 and γ = 0.57, *t* = 7.26, *p* < 0.001, respectively. In summary, both Hypotheses H2 and H3a were supported, which means there were positive effect of the transformational leadership on the organizational commitment and the organizational commitment on the OCB.

In addition, the product of the path coefficient of organizational commitment and OCB was 0.68 × 0.57 = 0.39, and the organizational commitment had mediating role played between the transformational leadership and the OCB. However, the relationship between the transformational leadership and the OCB was not significant, with γ = 0.13, *t* = 1.60, *p* > 0.05. Kenny et al. (1998) pointed out that there were three preconditions for a complete mediation, including independent variable, the intermediate variable (organizational commitment) and the dependent variable (OCB) have to have a significant association. Second, the intermediate variable and the dependent variables have to be significantly associated. Finally, when the mediator variable was put into the SEM model, the relationship between the independent variables and the dependent variables should become insignificant. If the relationship between the independent variables and the dependent variables weakened while the mediator variable was still significant, it had only part of the mediating role. It can be found from Table 1, and from the statistical analysis, that organizational commitment played a complete mediating role. Therefore, Hypothesis H3b was supported. The aforementioned results were consistent with the existing research results. That means transformational leadership and organizational commitment had positive significant effect on the organizational commitment and OCB, and organizational commitment had a mediating role played between the transformational leadership and the OCB relationship (Hamidi and Salimi, 2015; Indarti et al., 2017; Eliyana and Ma'arif, 2019).

#### Hierarchical linear modeling tests

##### The null model

In this study, a null model was established to test whether the relationship between the group-level and the individual-level variables, and the reveal of the OCB of immigration officers had reached a significant level. It was used to determine whether the significant differences were achieved between the groups to which each subject belongs (Kuonath et al., 2017). From Table 4, it could be found that there were differences between the groups, with τ_00_ = 0.083, *df* = 24, Wald *Z* = 3.243, *p* < 0.001, which showed that the “OCB” was different between the subjects.

**Table 4:** Hierarchical linear modeling results for individual variables.

**Variable**	***γ***_**01**_	***τ***_**00**_		***γ***_**11**_
1. The null model		0.094^***^	3. Moderating effects (slopes-as-outcomes model)	
2. Context effects (intercepts-as-outcomes model)			(5) Aggregated transformational leadership (Organizational commitment – OCB)	0.245^*^(0.231)
(1) Aggregated transformational leadership–organizational commitment	0.102 (0.101)		(6) Organizational climate (Organizational commitment – OCB)	0.227^*^(0.155)
(2) Aggregated transformational leadership–OCB	0.093 (0.097)			
(3) Organizational climate–organizational commitment	0.326^**^(0.137)			
(4) Organizational climate–OCB	0.457^***^(0.146)			

*The numbers in bracket are standard error; (1) to (4) are the contextual effects of group-level variables on individual-level variables. For example, aggregated transformational leadership–organizational commitment is the contextual effect of group-level aggregated transformational leadership on the individual-level organizational commitment; (5) to (6) are the moderating effect of group-level variables on the relationship between individual-level independent variables and the dependent variable. For example, aggregated transformational leadership (organizational commitment–OCB) moderates the relationship between the individual-level independent variables (organizational commitment) and the dependent variable (OCB). The table lists the indicators for tested hypotheses only*.

* *p < 0.05;*

** *p < 0.01;*

*** *p < 0.001*.

*The bold values indicate the test indicators for each hypothesis*.

##### Contextual effects (intercepts-as-outcomes models)

This study has applied the hierarchical linear model to conduct “intercepts-as-outcomes models” for the organizational commitment and the OCB to explain the incremental variation at level 1. Besides, it adopted group-level transformational leadership and organizational climate as the explanatory variables of incremental variation at level 2 (Shen, 2016). This study has applied γ_01_ parameter to estimate whether group-level variables had contextual effect on individual-level variables to predict whether aggregated transformational leadership and organizational climate had positive effect on individual level of the organizational commitment and the OCB. It was the aim to inspect whether Hypotheses H4a and H4b; and Hypotheses H5a and H5b were supported, respectively.

Table 4 showed that the aggregated transformational leadership had no effect on the organizational commitment and the OCB, and their relevant indicators were γ_01_ = 0.102, SE = 0.101, *t* = 1.34, *p* > 0.05 and γ_01_ = 0.093, SE = 0.097, *t* = 1.23, *p* > 0.05, respectively. In addition, the organizational climate had significant effect on the organizational commitment and the OCB, and their relevant indicators were γ_01_ = 0.326, SE = 0.137, *t* = 2.08, *p* < 0.05 and γ_01_ = 0.457, SE = 0.146, *t* = 3.85, *p* < 0.001, respectively. Therefore, the organizational climate had cross-level effect on the individual-level variables, but aggregated transformation leadership did not. As stated above, Hypotheses H4a and H4b were supported, whereas Hypotheses H3a and H3b were not supported. The results showed that under the multi-level organizational structure of Taiwan NIA, the individual-level dependent variables could be affected by the group-level variables. It meant that they would be more confident in their work and reveal more OCB when individual understood the higher levels of organizational climate.

Besides, the differences in perception of organizational atmosphere climate among would also affect the level of performance of employees in organizational commitment and OCB (Kao, 2017).

##### Moderating effects (slopes-as-outcomes model)

The relevant studies found that the random variance of organizational commitment in the intercept-as-outcomes model reaching significant would be the prerequisite for its moderating effect (Walumbwa et al., 2008). The test found that the random variance of aggregated transformational leadership and organizational climate on organizational commitment were 0.0011 (*p* < 0.05) and 0.008 (*p* < 0.05), respectively. That is, the aggregated transformational leadership and organizational climate had reached significant differences on relationship between the organizational commitment and the OCB of NIA employees. After this premise has been confirmed to test whether Hypotheses H6 and H7 of this study were supported, the explanatory variables of group-level variables were applied to evaluate the explanatory power of the variables. The test results were shown in Table 4. The aggregated transformational leadership predicted a significant slope between the organizational commitments and OCB relationships. The indexes were γ_11_ = 0.227; *t* = 1.975, *p* < 0.05. Thus, Hypotheses H6 and H7 were supported.

According to the above results, we could find that when the supervisor's leadership is transformational leadership by the employee's perception, the employees are more likely to show a higher level of commitment and indirectly promote more OCB. As a result, a leader of the immigration team should establish an idea based on a common theme for their employees to believe and accept the organization goals and values as well as to act for the interest of the organization. Meanwhile, to elevate frontline the immigration organization's commitment and OCB, a supervisor should clearly and concisely express the vision of the organization to encourage all levels within the organization to do their best for their work and to correctly understand the organization's goals to promote the employees to carry out more citizenship behavior benefiting the organization and clients. This result also indicates that in the immigration organization, what is perceived overall by the employees about their leaders' transformational leadership can positively affect organizational climate perceived by employees overall. Therefore, the leaders of the immigration organizations should clearly express the vision of their organizations, encourage their subordinates to solve problems from new perspectives, and care about each employee individually to help the subordinates develop affection for the organization and to approve the organization. By doing so, the employees will feel positively about organizational climate.

In addition, the findings of this study shows that a leader of an immigration officer should show the vision of the organization and communicate with the subordinates on methods and strategies to achieve the organizational goals, and promote a good organizational climate in order to encourage the immigration officers to act for the interest of the organization (Moslehpour et al., 2019).

This study found that there is a special situation in which the relationship between aggregate–transformative leadership on an individual's organizational commitment and OCB manifestations has a cross-level interaction, but no contextual effect. Since Taiwan NIA has been regarded as a bureaucratic paramilitary institution (Cho, 2017), this study believed that it may be caused by its special organizational structure and the cost. Moreover, this phenomenon can also be generated by attentively listening to the immigration officers' opinions and having transformational leader act as a supervisor or coach who cares about employees individually and pays attention to their achievement and needs for development. Therefore, compared to the overall group effect, the leader has a more direct and concrete effect on individual follower, and compared with other organizations, the leaders of Taiwan NIA organizations could more directly pass on their vision of their subordinates, or personal care and inspiration, to their subordinates through the hierarchical relationship of the organization. This may be the reason why it was more convenient for the supervisors to reveal the transformational leadership at the individual level, and thus it made the transformational leaders to have a greater effect on the organizational commitment and on the OCB.

## Conclusion and suggestion

### Conclusion

According to the analysis, this study found that the aggregated that the transformational leadership had significant effect on organizational climate. This is consistent with existing research results (e.g., Kohan et al., 2018). Moreover, according to SEM analysis, it is found that the transformational leadership of organizations in Taiwan NIA had positive effect on organizational commitment of employees, and affected the individual reveal of OCB. Simultaneously, the organizational commitment also had good and positive effect on individual reveal of OCB. Furthermore, after the HLM analysis, this study found that the aggregated transformational leadership had no cross-level contextual effect on individual level of dependent variables, but the organizational climate did. This result showed that organizational climate could form the shared values of the group and individual members (Martinolli et al., 2021), and encourage them to hang together (Kao, 2017). Besides, a good organizational climate could be used by allowing the employees to appreciate the care or concern of the organization for them, and to build optimistic, cheerful, and lively. It did not only make members to feel that they were important to the team, but it could stimulate their organizational commitment and actively reveal OCB. The organization could be benefited from it. After the HLM test to verify Hypotheses H5 and H6, this study found that the aggregated transformational leadership and the organizational climate could have a cross-level interactive effect on individual reveal of OCB through the additive effect of organizational commitment. Based on these results, it also showed that the aggregated transformational leadership of the organization executives of the Taiwan NIA was effective and the organizational climate was positive.

### Theoretical and practical implications

The study stresses on some key features of HLM for analyzing the leadership or the organizational climate focused hierarchical research. The result of this study revealed three important advantages of HLM in analyzing the multi-level immigration organization. First, the HLM is a very valuable statistical technology for interpreting multi-level data, including those already demonstrated ones (Bryk and Raudenbush, 1992). Second, the HLM reveals a more in-depth look of the organization behavior because for the variances of different sources in outcome variables, the HLM can be used for identifying and verifying the source of variances, and at different analysis levels, the HLM offers an operation method using multiple explanatory variables (Gavin and Hofmann, 2002). Third, for evaluating the contextual effects and cross-level interactive effects, the HLM provides a forceful and potent measurement. Based on the theoretical implications of the HLM, this study reinterprets the direct, cross-hierarchical context and interaction of aggregate transformational leadership and organizational atmosphere on individual-level variables so that the influence of group power in the organizations can be concretely presented. Even though the researchers have already extensively showed that the leadership and the organizational climate are critical factors affecting the employee behavior. However, when the group-level variables were included in this study, it was found that the transformational leadership of the supervisors and the organizational climate of the group have different structures in the causal relationship (individual level) that affects the employee behavior, that is, there will be cross-level effects. Therefore, for the conventional way of using individual-level perception and attitudes as factors for analysis, the group-level variables can actually offer us another research perspective and thinking (Kao, 2017).

In addition to the theoretical implications above, the following practical implications were also included.

First, the research finding suggested that the contextual effect and cross-level moderating effect of ATL and organizational climate on individual-level outcome variables should be examined. It is especially true for an immigration organization to be responsible for the border security and serve the domestic and foreign tourists.

Second, the new approach of stimulating subordinates for problem-solving, coping with challenges as well as of verifying the needs of the immigration organization enables the transformational leaders to stimulate the immigration officers to pay more attention on their work, and therefore a higher organizational commitment can be generated.

Third, the leaders of the Taiwan NIA organizations should establish a good and achievable vision to inspire and encourage their subordinates to take on responsibilities, to increase employee commitment to the organization.

Fourth, if the transformational leaders could create a positive, uplifting, and pleasant climate, and empower subordinates with confidence, leaders could increase the organizational commitment of subordinates accordingly. Thus, the supervisors of Taiwan NIA organizations should create a pleasant climate for the immigration officers and make them to be willing to work hard for the organization. This would develop more OCB that would benefit the organizations.

Fifth, explaining the goals, using clear and definite descriptions on the service role of the immigration officer, and the reward system can encourage frontline subordinates to display the OCB voluntarily (e.g., the immigration teams). In addition, the recruitment and selection practice is a great opportunity for attracting and choosing OCB-oriented employees (Salas-Vallina et al., 2021). Therefore, the immigration organization should be aware of the above suggestions and work on understanding if their staff is service oriented.

Sixth, since the transformational leadership could affect the organizational commitment and the OCB performance of the subordinates, the managers of all levels of Taiwan NIA organizations should adopt the leadership styles that give employees a vision and inspire their subordinates wisdom and inspiration so that Taiwan NIA could be successful implement the leadership effectiveness. In addition, based on the overall operation of the Taiwan NIA, the leader of the immigration organizational should fully reduce “powerful character of the immigration organizational socialization process” (Metcalfe and Dick, 2002, p. 396), respond to the service needed of the public, and eliminate unnecessary bureaucratic delay (Adebayo, 2005).

Finally, based on the appropriate human resource management practices, employee promotion can demonstrate positive organizational behavior, and new management thinking and technology have been gradually applied in employee management practices. This study suggests that Taiwan's NIA can use workforce analysis in employee management to enhance the organization's human resources, and the correctness of its use. Moreover, to adopt new technologies to diagnose employee deficiencies and adequate human management practices, it can significantly improve an organization's ability to achieve its goals (Olsen, 2019). For example, using the Internet of Things and artificial intelligence (Pera, 2019), big data analysis, and decision automation to optimize employee performance (Bacalu, 2021).

## Data availability statement

The original contributions presented in the study are included in the article/supplementary material, further inquiries can be directed to the corresponding author.

## Ethics statement

The studies involving human participants were reviewed and approved by the Ministry of Science and Technology, Taiwan (R.O.C). Written informed consent to participate in this study was provided by the participants.

## Author contributions

C-CC and R-HK: conceptualization. C-CC: methodology, software, investigation, and resources. R-HK: validation and formal analysis. All authors contributed to the article and approved the submitted version.

## Conflict of interest

The authors declare that the research was conducted in the absence of any commercial or financial relationships that could be construed as a potential conflict of interest.

## Publisher's note

All claims expressed in this article are solely those of the authors and do not necessarily represent those of their affiliated organizations, or those of the publisher, the editors and the reviewers. Any product that may be evaluated in this article, or claim that may be made by its manufacturer, is not guaranteed or endorsed by the publisher.
